# Pyeloduodenal Fistula in Xanthogranulomatous Pyelonephritis

**DOI:** 10.1093/jcag/gwac008

**Published:** 2022-03-16

**Authors:** Grace Wang, Parul Tandon, Christopher Wayne Teshima

**Affiliations:** Department of Gastroenterology and Hepatology, University of Toronto, Toronto, Canada; Department of Gastroenterology and Hepatology, University of Toronto, Toronto, Canada; Department of Gastroenterology and Hepatology, University of Toronto, Toronto, Canada

A 48-year-old otherwise healthy woman with recurrent urinary tract infections presented to the emergency department with 1 month of fever and flank tenderness. Three months prior, she was diagnosed with a 3-cm right staghorn stone; interventions were postponed due to the SARS-CoV-2 pandemic. Percutaneous nephrolithotomy was attempted: unexpectedly, dye injected into the ureter filled the bowel on fluoroscopy, suggesting a renal-enteric fistula (a). Computed tomography demonstrated xanthogranulomatous pyelonephritis (XGP), a rare chronic pyelonephritis, with perinephric stranding abutting a thickened duodenum. Endoscopy was performed to plan for intervention, revealing a fistulous opening in the second segment of the duodenum (b). Pyeloduodenal fistula is an extremely rare, late-presenting sequela of XGP (1). Definitive management traditionally involves primary surgical fistula closure with nephrectomy (2). Endoscopy facilitates fistula localization; furthermore, with the advent of Over-the-Scope-Clips (OTSC) for fistula closure, endoscopic approaches should be considered first as a safe, feasible alternative to surgery. There are two reported cases of pyeloduodenal fistula closure using OTSC (3,4). Endoscopic intervention was considered here; however, given the patient’s significant renal involvement, she underwent nephrectomy with concurrent surgical clipping of the fistulous tract. Pathology revealed abscesses and necrosis infiltrating the renal parenchyma. Post-operatively, she required parenteral antibiotics and remained well 6 months post-discharge.


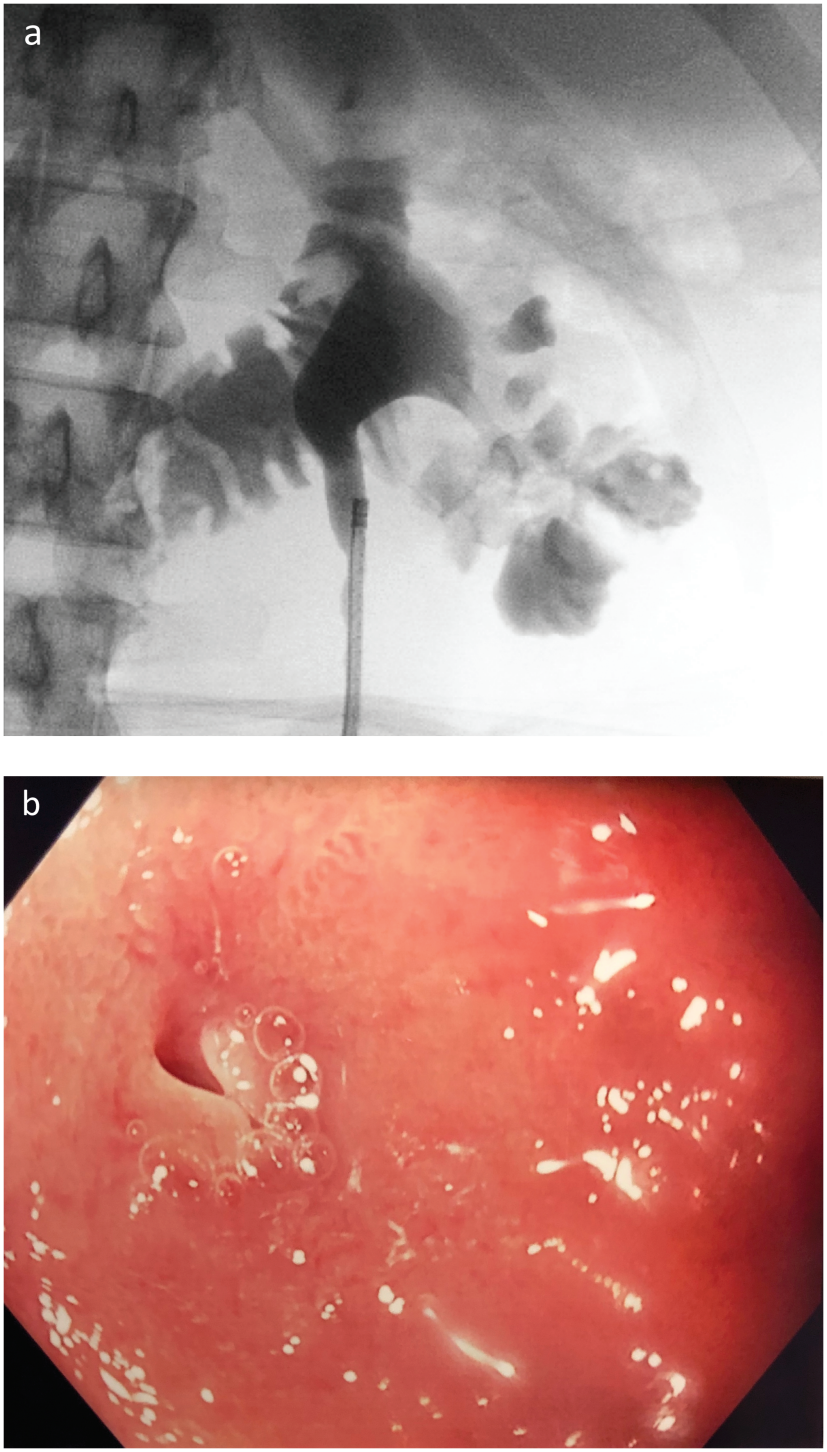


## References

[1] Cohen MH , Becker MH, Hotchkiss RS. Pyeloduodenal fistula: Report of a case and review of the literature. J Urol 1966;95:678–80. 5935528 10.1016/S0022-5347(17)63517-1

[2] Rodney K , Maxted WC, Pahira JJ. Pyeloduodenal fistula. Urologys 1983;22:536–9. 10.1016/0090-4295(83)90237-6 6649212

[3] Aslam B , Frandah W, Mardini H. A novel use of over-the-scope clip for management of duodenal-renal enteric Fistula. Am J Gastroenterol 2018;113(Supplement):S1199–200.

[4] Duh E , Clary M, Samarasena J, Clayman RV, Chang K. Successful endoscopic closure of pyeloduodenal fistula using an over-the-scope clip. ACG Case Reports Journal 2019;6(11):e00281. 32309478 10.14309/crj.0000000000000281 PMC7145216

